# Don’t turn your back on the symptoms of psychosis: a proof-of-principle, quasi-experimental public health trial to reduce the duration of untreated psychosis in Birmingham, UK

**DOI:** 10.1186/1471-244X-13-67

**Published:** 2013-02-22

**Authors:** Charlotte Connor, Max Birchwood, Colin Palmer, Sunita Channa, Nick Freemantle, Helen Lester, Paul Patterson, Swaran Singh

**Affiliations:** 1 CLAHRC, Birmingham & Solihull Mental Health Foundation Trust, 66-68 Hagley Road, B16 8PF, Birmingham, UK; 2 School of Psychology, University of Birmingham, Edgbaston, B15 2TT, Birmingham, UK; 3 Youth Mental Health, School of Psychology, University of Birmingham, Edgbaston, B15 2TT, Birmingham, UK; 4 Youthspace Mental Health Service, Birmingham and Solihull Mental Health Foundation Trust, Edgbaston, B15 2TT, Birmingham, UK; 5 Clinical Epidemiology & Biostatistics, Department of Primary Care and Population Health, UCL Medical School (Royal Free Campus), Rowland Hill Street, NW3 2PF, London, UK; 6 Primary Care, Primary Care Clinical Sciences, School of Health and Population Sciences, Primary Care Clinical Sciences Building, University of Birmingham, Edgbaston, B15 2TT, Birmingham, UK; 7 Youthspace, Birmingham & Solihull Mental Health Foundation Trust, 66-68 Hagley Road, B16 8PF, Birmingham, UK; 8 Mental Health & Wellbeing, Medical School Building, Gibbet Hill Campus, University of Warwick, CV4 7AL, Coventry, UK

**Keywords:** Public mental health campaign, First-episode psychosis, Early detection, Duration of untreated psychosis, Youth mental health

## Abstract

**Background:**

Reducing the duration of untreated psychosis (DUP) is an aspiration of international guidelines for first episode psychosis; however, public health initiatives have met with mixed results. Systematic reviews suggest that greater focus on the *sources* of delay within care pathways, (which will vary between healthcare settings) is needed to achieve sustainable reductions in DUP (BJP 198: 256-263; 2011).

**Methods/Design:**

A quasi-experimental trial, comparing a targeted intervention area with a ‘detection as usual’ area in the same city. A proof-of–principle trial, no a priori assumptions are made regarding effect size; key outcome will be an estimate of the potential effect size for a definitive trial. DUP and number of new cases will be collected over an 18-month period in target and control areas and compared; historical data on DUP collected in both areas over the previous three years, will serve as a benchmark. The intervention will focus on reducing two significant DUP component delays within the overall care pathway: delays within the mental health service and help-seeking delay.

**Discussion:**

This pragmatic trial will be the first to target known delays within the care pathway for those with a first episode of psychosis. If successful, this will provide a generalizable methodology that can be implemented in a variety of healthcare contexts with differing sources of delay.

**Trial registration:**

http://www.controlled-trials.com/ISRCTN45058713

## Background

International studies have shown that the average length of time from the onset of psychotic symptoms to first treatment (duration of untreated psychosis; DUP) ranges between 364 and 721 days [1] with recent meta-analyses reporting an association between long DUP and poor outcome at 6 and 12 months, for both symptoms and quality of life. Drake et al. [2] suggest that the point at which DUP *exceeds* three months, defines a critical point after which the likelihood of poorer outcome begins to increase, and several studies indicate that accessing treatment *within* the first 6 months of onset is a key indicator of outcome, specifically in terms of treatment response [3-8].

Evidence for the efficacy of initiatives to reduce DUP, however, is mixed [9]; the ‘TIPS’ study in Norway has had the greatest success. Their prospective trial in a defined health care region, incorporating the introduction of an early detection program alongside a public health awareness campaign, achieved significant results in comparison with parallel health care areas *without* an early detection program. DUP was significantly shorter and associated with better clinical status and reduced suicide risk at baseline and negative symptoms at 12 months, with positive effects on clinical and functional status maintained at 5 year follow-up [10]. However, similar public health initiatives in Australia [11] and Canada [12] have failed to demonstrate any impact on DUP.

Implementation of a TIPS style intervention in UK healthcare settings, however, may prove ineffective as the care pathway delays experienced by young people with first-episode psychosis in UK may not be directly comparable to those experienced in Norway. Rogaland County is a dispersed population centred around the predominantly Caucasian (95%) city of Stavanger [13]; approximately 60% of the population living in urban areas [14]. In contrast, Birmingham, UK is the second most populous city in Britain (1.05m) and the ‘youngest city in Europe’ with 40% of its population under the age of 20. It has a high degree of cultural and religious diversity, including 68% White British residents, 20% residents of Asian or Asian British heritage and 7% Black or Black British, and includes the highest number of residents from the Muslim faith in any UK local area for whom help-seeking can include imams at the family mosque; indeed, our recent study showed that religious construction of psychosis is common [15].

In the UK, the widespread adoption of early intervention in psychosis teams (EIS) have not led to a reduction in DUP [16,17]; this is, perhaps, not surprising since these teams are not resourced or equipped with any *community focused* early detection function. Birmingham, UK, was the setting of the first early intervention service in the UK, yet despite this it continues to experience long DUP [18]. Our cluster randomized trial to improve early detection of psychosis focusing on primary care [16] did not achieve reductions in DUP; further analysis revealed that the main sources of delay occurred elsewhere in the care pathway, in particular, *within* mental health services themselves and in help-seeking delay ^16; 18^. A recent systematic review of interventions to reduce DUP [1] concluded that greater focus on the *sources* of delay within care pathways, (which will vary between healthcare settings) is needed to achieve sustainable reductions in DUP.

Recent data from the UK National EDEN study [19] has confirmed that there are two primary sources of delay: delay in help-seeking among both patients and carers and delays within mental health services. The first referral point to mental health services was found to have the greatest impact on DUP: where the first point of contact was for acute crisis (admission, home treatment), delay within the mental health service was low; where access was via a Community Mental Health Team (CMHT), delays were greatly extended. These findings suggested that whilst improvements in help-seeking behavior are necessary, significant improvements within mental health services themselves would be essential to reduce DUP. In Birmingham, as part of the present study, a youth access pathway into the mental health service has been developed within the CMHT [20], providing a single referral point and ensuring that first episode cases of psychosis are managed in a youth sensitive framework and guarantee direct access to the specialised service.

### Hypothesis

The primary hypothesis to be tested is whether implementation of a psychosis public health campaign *in addition to* the youth access pathway for first episode psychosis, will significantly reduce DUP.

## Methods

### Design

This is a quasi-experimental, proof-of-principle prospective trial comparing an intervention area in the south of the city, to the non-intervention remainder of the city.

Incident cases of first episode psychosis from these areas will be determined and their DUP and care pathways ascertained over the following 18 months. We also benefit from recent historical DUP data (National EDEN) [19] for both target and control areas.

### Sampling

Birmingham is served by a single mental health service, which served as the ‘blueprint’ for the Government’s National Service Framework for Mental Health [21]; it has a number of community services including Home Treatment and Assertive Outreach teams, including well-developed early intervention services for first episode psychosis which were the forerunner of such services across England. It is the second most populous city in Britain with a high degree of cultural and religious diversity and is ranked third most deprived core city in England [22]. Population and ethnic profile of the intervention and control areas [23], together with the number of new cases in the previous 12 months, is shown in Table 1.

**Table 1:** Socio-demographic profile of intervention and control areas

	**Population**	**Ethnic profile**
**Intervention Area**	308,150	85% White British
		8% Asian
		3% Black
**Control Area**	632, 427	62% White British
		26% Asian
		8% Black

### Ethical approval

The trial is sponsored by Research and Innovation department at Birmingham & Solihull Mental Health Foundation Trust. Advice was sought from the National Research Ethics Service for the NHS. This public health trial falls outside of the requirements for formal approval as: no consent or recruitment of service users is required; no identifiable data will be specifically collected or used for evaluation; and the only data to be used will be anonymised DUP data and number of incident cases, both of which are routinely collected for all clients entering the specialist Early Intervention Service (for first episode psychosis) as part of their initial assessment.

### The intervention

#### Context and theoretical framework

The ‘Precede-Proceed’ public health model framework [24] has provided the foundation for this study using assessments of context and setting to inform and guide the development and implementation of the intervention. This will ensure a responsive, stratified ‘knowledge-transfer’ approach is applied to reducing DUP. This framework is further underpinned by two theoretical models of health behaviour change addressing both cognitive *and* contextual determinants; the Trans-theoretical/Stages of Change model [25] and the MINDSPACE framework [26]^,^ the latter arising from behavioural economics, widely employed by UK policymakers [27].

Initial findings from the ‘Precede’ phase of our programme have helped us clarify the likely reasons for delay in care pathways and enabled comprehensive assessment, planning, piloting and target-setting [18]. Patient and public involvement was a fundamental aspect of this preceding phase, involving discussion and consultation with an advisory board comprising of young people and users of the mental health services.

The ‘Proceed’ phase now focuses directly on implementation and evaluation of the intervention, but will, nevertheless, continue to incorporate comprehensive and iterative feedback at each stage to determine the most successful methods of improving help-seeking and youth care pathways.

#### Care pathway interventions

1. Improving help-seeking.

The aim of this component of the intervention is to improve the help-seeking of young people and their carers, who are experiencing symptoms of first-episode psychosis through implementation of a psychosis awareness campaign including information about when, where and how to seek help. Individuals are encouraged to access a helpline and a bespoke website.

The campaign was launched in January 2012 and will run for 18-months. Campaign staff, will be active at the heart of the community, in local shopping centres and supermarkets, at employment centres and community events and will work closely with youth and community groups. All campaign material bears the psychosis campaign slogan, ‘*Don’t turn your back on the symptoms of psychosis’*, the website link (http://www.youthspace.me/psychosis) and the information line number.

The campaign will be broken into 6 three-month stages. Each stage comprising of the following elements:

i. Advertising in high use settings.

Campaign posters will be displayed in a variety of settings, including local bus services and shopping centres. Smaller leaflets and postcards will be routinely distributed across the target areas including supermarkets, employment offices, community and youth groups, leisure centres, coffee shops and fast-food outlets. Informed by our pathfinder studies, the poster themes focus specifically on young people, their friends and parents/carers.

ii. Leaflet drops

Leaflets drops to homes on several large social housing estates in hard-to-reach areas which have no central shopping centre, high street or supermarket.

iii. Advertising in community press

Posters and articles about psychosis, featuring interviews with clinicians and service users, will appear in community newspapers and magazines at regular intervals throughout the campaign; these monthly, bi-monthly and quarterly newspapers and magazines are delivered free to homes in the target areas.

iv. Advertising on community websites

Posters, on-going campaign information and articles about psychosis will be routinely posted on 6 community websites and 10 library web-pages in the target area.

v. Attendance at community events

Campaign volunteers and staff will attend a variety of community and music events and also attend student events at Universities and colleges in the intervention area.

vi. *Promotion of*http://www.youthspace.me/psychosis

Launched in early 2011, our website offers advice, resources, signposting, educational films, blogs and social media access for young people, families/carers and those who work alongside young people on all aspects of mental health and well-being. For the purposes of the psychosis campaign, a direct link to the psychosis specific page of the website will be promoted, providing a variety of information about psychosis, the benefits and importance of early help-seeking, and where to go to seek help.

vii. The Psychosis Information line

A psychosis information line features on all campaign material, offering an alternative way of help seeking for those who would rather *speak* to trained advisors about their concerns or for those who have no access to a computer. A similar line was utilised in the TIPS study. Its main function will be to provide callers with information about psychosis, send out information packs and facilitate access to a clinician for further assessment where appropriate.

viii. Youth Advisors

Youth advisors will regularly update the campaign with photographs, video footage and blogs by those who have experienced mental health issues and ensure we utilise timely and age appropriate creative strategies to engage with young people.

ix. Psychosis Awareness Training

We will collaborate with Emergency Services, youth, community, employment and education agencies to provide psychosis awareness training; this will enable greater outreach into the community and create a broad network of organisations and individuals through which the campaign can be targeted.

2. Youth mental health care pathway.

A youth access care pathway was developed operating alongside existing CMHTs in the intervention area and provides direct referral channels and immediate assessments for all young people presenting to primary care with mental health difficulties aged 14–25 [20]. The service was established to improve the care pathways of young people ensuring that all cases of first episode psychosis have direct access to EIS, enabling and maintaining good levels of engagement with young people from their earliest presentation.

### Inclusion and exclusion criteria

All new cases of first episode psychosis accepted by the Early Intervention in psychosis Service.

Patients considered at ultra-high risk of psychosis, using the Comprehensive Assessment of At Risk Mental State (CAARMS) [28] are excluded. Since this is a pragmatic trial focussing on reducing DUP in patients managed within an EIS, there will be no other exclusions.

### Measures

DUP is routinely calculated for all clients with a first episode of psychosis at entry into EIS services, employing a combination of retrospective assessment of positive and negative symptoms of psychosis (SCI-PANSS) [29], Pathways to Care interview [30] and electronic care records. This is based on the method described by Larsen et al [10] and used in our research [18].

The Pathways to Care interview follows the method of Gater et al. [30] in which systematic information is gathered from direct interview and electronic care records about the source, sequence and timing of help-seeking by patients and their families, including help-seeking contacts, the main problems presented and treatments offered. This included the sequence and duration of contacts within the mental health service. We also documented any mental health service contact prior to the formal onset of psychosis. These data were synthesised onto visual ‘route timelines’; presenting the sequence of help-seeking contacts, referrals made, diagnoses offered, treatment provided and outcomes.

All DUP interviews are conducted by graduate research psychologists, who have satisfactorily completed five DUP calculations, reproducing scores pre-defined by trainers [18]. Each interview takes approximately 1 hour to complete and research staff undergo 6-monthly checks on their assessment reliability, consisting of submission of five timelines and DUP calculations to DUP co-ordinators for concordance and standardisation of calculation. Continual feedback and assistance from co-ordinators was maintained throughout data collection to ensure inter-rater reliability.

### Primary outcome

#### DUP

We hypothesise that our psychosis awareness campaign, *in combination with* the introduction of the youth focused clinical access team, will significantly reduce DUP.

### Secondary outcome

#### Referral rates

We also predict an *increase* in referrals of young people diagnosed with a psychotic disorder from the intervention area into EIS.

Table 2 shows baseline DUP and component delays for incident cases in between August 2011 and August 2012.

**Table 2:** Number of incident cases and their DUP: August 2011 – August 2012

	**New cases**	**DUP (mean; median; sd)**	**Delay in help-seeking (mean; median; sd)**	**Delay within mental health services (mean; median; sd)**	**Delay in accessing EIS (mean; median; sd)**
**Intervention Area**	88	293; 38 (867.1)	50; 0 (131.6)	211; 30 (481.4)	443; 56 (930)
**Control Area**	250	264; 58 (539)	114; 3 (271.4)113	213; 22 (511.9)	323; 105 (560.8)

### Primary analysis

DUP and referral rates for intervention area compared to the control area will be monitored and evaluated every three months throughout implementation and at the culmination of the intervention (2 year period). In the TIPS study, despite DUP being relatively short in both areas (with a combined median of 10 weeks), DUP was significantly reduced in the intervention area, reduced to a median of 5 weeks compared with 16 weeks in the control area [31]. In this proof-of-principle trial, we make no a priori assumptions about effect size since the methodology and setting is unique. The key outcome of this trial will be an estimate of the effect size, which will inform a definitive trial. The confidence intervals for different levels of increase in the number of new referrals in the target area are shown in Table 3.

**Table 3:** Confidence Intervals for the target area based on predicted increase in referral rate

**Yearly referral rate increase**	***Predicted *confidence Interval (+/−)**	**CI width**
30	218	90-526
40	187	121-495
50	166	142-474
**60**	**151**	**157-459**
70	139	169-447
80	130	178-438
90	123	185-431
100	116	192-424

### Secondary analysis

#### Website visits

We also predict there will be an increased number of online visits to our website http://www.youthspace.me/psychosis. The website went live in September 2011. Website visits will be monitored on a monthly basis before, during and after the intervention.

#### UK benchmark DUP data

The trial benefits from extensive DUP data from UK National EDEN sites in Cambridge, Cornwall, Blackburn, Blackpool, Burnley, Lancaster and Norwich [18].

## Discussion

Although reducing DUP is a UK Department of Health target, there has been no effective strategy for achieving this in the UK. This is the first UK trial attempting to reduce DUP. The ethnic profile of Birmingham, however, is diverse and includes large numbers of inhabitants of Asian and Black heritage, therefore, any findings from this intervention, will be need to be carefully considered in light of this diversity. Our application of the Precede/Proceed public health model in the design and implementation of the trial, nonetheless, will provide a *generalizable* methodology that should be applicable to a variety of healthcare contexts with differing sources of delay.

## Competing interests

The authors declare that they have no competing interests.

## Authors’ contributions

The trial was designed by the first two authors; all authors read and approved the final manuscript.

## Pre-publication history

The pre-publication history for this paper can be accessed here:

http://www.biomedcentral.com/1471-244X/13/67/prepub
